# Suppression of long-branch attraction artefacts in the animal phylogeny using a site-heterogeneous model

**DOI:** 10.1186/1471-2148-7-S1-S4

**Published:** 2007-02-08

**Authors:** Nicolas Lartillot, Henner Brinkmann, Hervé Philippe

**Affiliations:** 1 Laboratoire d'Informatique, de Robotique et de Microélectronique de Montpellier, UMR 5506, CNRS-Université de Montpellier 2, 161, rue Ada, 34392 Montpellier Cedex 5, France; 2 Canadian Institute for Advanced Research, Département de Biochimie, Université de Montréal, Montréal, Québec Canada

## Abstract

**Background:**

Thanks to the large amount of signal contained in genome-wide sequence alignments, phylogenomic analyses are converging towards highly supported trees. However, high statistical support does not imply that the tree is accurate. Systematic errors, such as the Long Branch Attraction (LBA) artefact, can be misleading, in particular when the taxon sampling is poor, or the outgroup is distant. In an otherwise consistent probabilistic framework, systematic errors in genome-wide analyses can be traced back to model mis-specification problems, which suggests that better models of sequence evolution should be devised, that would be more robust to tree reconstruction artefacts, even under the most challenging conditions.

**Methods:**

We focus on a well characterized LBA artefact analyzed in a previous phylogenomic study of the metazoan tree, in which two fast-evolving animal phyla, nematodes and platyhelminths, emerge either at the base of all other Bilateria, or within protostomes, depending on the outgroup. We use this artefactual result as a case study for comparing the robustness of two alternative models: a standard, site-homogeneous model, based on an empirical matrix of amino-acid replacement (WAG), and a site-heterogeneous mixture model (CAT). In parallel, we propose a posterior predictive test, allowing one to measure how well a model acknowledges sequence saturation.

**Results:**

Adopting a Bayesian framework, we show that the LBA artefact observed under WAG disappears when the site-heterogeneous model CAT is used. Using cross-validation, we further demonstrate that CAT has a better statistical fit than WAG on this data set. Finally, using our statistical goodness-of-fit test, we show that CAT, but not WAG, correctly accounts for the overall level of saturation, and that this is due to a better estimation of site-specific amino-acid preferences.

**Conclusion:**

The CAT model appears to be more robust than WAG against LBA artefacts, essentially because it correctly anticipates the high probability of convergences and reversions implied by the small effective size of the amino-acid alphabet at each site of the alignment. More generally, our results provide strong evidence that site-specificities in the substitution process need be accounted for in order to obtain more reliable phylogenetic trees.

## Background

With the advent of genomic sequence data, phylogenetics is progressively switching to large-scale analyses, using many genes in parallel [1]. Among the diverse methods that have been proposed for dealing with multigene data sets is the so-called supermatrix method [2]. This method consists in concatenating the sequences of all available genes into one single "supergene", which is then subjected to standard phylogenetic reconstruction methods. An obvious advantage of relying on large sequences is the expected increase of the statistical support; as long as all or most of the genes included in the analysis have an evolutionary history congruent with that of their host species (i.e. in the absence of hidden paralogies or lateral gene transfers), the small amounts of phylogenetic signal contained in each gene should in principle add up and overwhelm stochastic errors, thus leading to a well-supported phylogenetic tree.

However, high statistical support does not necessarily imply that the trees obtained are correct. In some cases, in particular under poor taxon sampling [3-6], highly resolved trees have been proposed, which have nevertheless been followed by subsequent critical re-analyses, claiming that, however strongly supported, the trees obtained were probably wrong [7-9]. More generally, there are several cases where standard phylogenetic reconstruction methods yield wrong but statistically well-supported trees. These so-called systematic (as opposed to stochastic) errors have been known about for a long time in the field [10,11], and are expected to be also present, in fact even enhanced, in a phylogenomic context [1,12].

A first explanation of systematic errors in phylogenetics is that they are caused by the mutational saturation of the sequences: if some positions have undergone multiple substitutions, this will blur the phylogenetic signal, and thereby increase the probability for several species to display convergent sequence patterns (homoplasies) at those positions. Many reconstruction methods are not able to correctly identify these convergences, and will instead interpret them as shared derived characters. As a consequence, they will be misled towards reconstructing a wrong tree. A typical instance of this phenomenon, called the long branch attraction (LBA) artefact [10], occurs when two phylogenetically distant species, evolving significanlty more rapidly than the rest of the taxa (hence have long branches), deceivingly appear as closely related in the estimated tree. Similarly, when a distant outgroup is used, a divergent species may be attracted by the long branch separating the in- and the outgroup, and thus be artefactually put at a basal position [11].

According to this explanation, removing the most saturated sites should improve the accuracy of the reconstruction. In this direction, several methods have been proposed, for selecting less diverged sequences [9,13], or filtering out saturated sites [14]. In most cases, these methods seem to bring a significant improvement. In addition, they are particularly advantageous in a phylogenomic context, where the amount of data is not limiting: fairly stringent filtering criteria can be applied, removing a large amount of data, but still leave behind a more than sufficient amount of phylogenetic signal to obtain well resolved trees [1].

An alternative way to deal with LBA artefacts is to avoid long branches altogether [12]. For instance, one can simply eliminate the fast-evolving taxa, and replace them by slow-evolving close relatives. This method was applied to the animal phylogeny, using 18S ribosomal RNA [15], and led to a reappraisal of the position of nematodes. Specifically, whereas fast-evolving nematodes, such as *Caenorhabditis*, would appear at the base of the group of bona fide coelomate Bilateria, a more slowly evolving one, *Trichinella*, appeared within arthropods. A related method consists in 'breaking' a long branch, by adding a series of intermediate taxa thought to emerge along this branch [16].

Altogether, a combination of a better taxon sampling and a more careful selection of sites or sequences makes it possible to converge to reliable phylogenies. And indeed, at most evolutionary scales (mammals [17], metazoans [9], plants [8], eukaryotes [18]), a consensus seems to be emerging regarding most evolutionary relationships in all these groups. On the other hand, such careful methods require significant expertise in phylogenetics. More fundamentally, they are exclusively focussed on the quality of the data, leaving open the problem of understanding why current reconstruction methods are so prone to systematic artefacts.

After all, LBA artefacts were initially described as an explanation of the statistical inconsistency of Maximum Parsimony (MP) [10], in contrast to probabilistic methods, which are consistent in a broad range of conditions [19,20]. As such, LBAs were expected to disappear once more reliable methods such as the Maximum Likelihood or the Bayesian frameworks became routinely used. Yet, artefacts are also observed under these methods, especially under poor taxon sampling. In a statistical perspective, the explanation of this apparent paradox is straightforward: the consistency property assumes that the underlying model is correct. Hence, such systematic errors simply betray that current models are mis-specified [21,22].

Note that explaining systematic artefacts as a model violation problem, as we do now, rather than one of mutational saturation, or of taxon sampling, as we did above, are not mutually exclusive arguments. When the data are not or are weakly saturated, current models (and also the MP method), all lead to the correct topology. It is only when the data are more strongly saturated, and the taxon sampling is not sufficiently rich to reveal the true extent of saturation, that a good model becomes necessary: in such situations, only the model can correctly estimate the frequency of multiple substitutions across the alignment, thereby avoiding systematic errors. Thus, what phylogenetic artefacts betray is fundamentally a lack of robustness of current models. More specifically, it points to the inherent propensity of these models to under-estimate the true level of saturation.

Many directions have already been explored to improve phylogenetic models, by accounting for compositional biases [23,24], across site heterogeneities of the rates [25,26], or of the substitution processes [27-32], or by acknowledging the variation of site-specific rates with time [33], non-independence between sites [34-36], etc. Some of these models have indeed resulted in improved phylogenetic inference [13,21]. In the present work, we will focus on site-heterogeneities of the amino-acid replacement processes, which may have a particularly strong impact on the way the model evaluates sequence saturation. A striking feature that one readily observes when working with protein alignments is the biochemical specificity observed at each site: in spite of the fact that there are 20 amino acids, only 2 to 4 distinct residues are typically observed at a given variable column, suggesting that most positions undergo repeated substitutions among a very restricted subset of the amino-acid alphabet [37,38]. Obviously, this pattern has a direct bearing on the expected level of homoplasy, as convergent evolution towards the same amino-acid will be all the more frequent as few amino-acids are allowed at a given site. It is therefore crucial to correctly account for this fact in models of protein evolution that are to be used for phylogenetic reconstruction.

In this direction, we have previously developed a mixture model that accounts for across site heterogeneities in the evolutionary processes [31]. Thanks to a Dirichlet process device, implemented in a Bayesian Monte Carlo framework, this model, CAT, effectively clusters the columns of the alignment into biochemically specific categories, each of which is described by its own amino-acid profile of equilibrium frequencies. By Bayes factor evaluation, we have shown previously that CAT generally has a better fit than homogeneous models based on one single empirical substitution matrix, such as WAG [39], JTT [40], or even the most general site-homogeneous and time-reversible model (GTR) [31,41]. We now apply the CAT model to the bilaterian phylogenomic dataset of Philippe et al [1,9]. This dataset displays an interesting example of systematic artefact When analyzed with current models of evolution, depending on the outgroup, two highly supported, yet contradictory, phylogenetic positions are obtained for two fast-evolving phyla, nematodes and platyhelminths. This artefact offers an experimental protocol for testing alternative models of evolution. Specifically, a good model should not lead to contradictory results depending on the chosen outgroup. In the present work, we use this case study to compare the performance of CAT to that of a site-homogeneous model based on the WAG matrix [39].

## Results and Discussion

### Robustness of CAT against LBA

We analyzed the phylogenetic position of nematodes or of platyhelminths as a function of both the outgroup and the evolutionary model. The dataset of Philippe et al. [9] was randomly cut into two halves, Meta1 and Meta2, which were analyzed in parallel. Apart from their specific amino-acid replacement processes, the two models under investigation, CAT and WAG, share the same features, including gamma-distributed rates across sites [25] (see methods).

**Figure 1:**
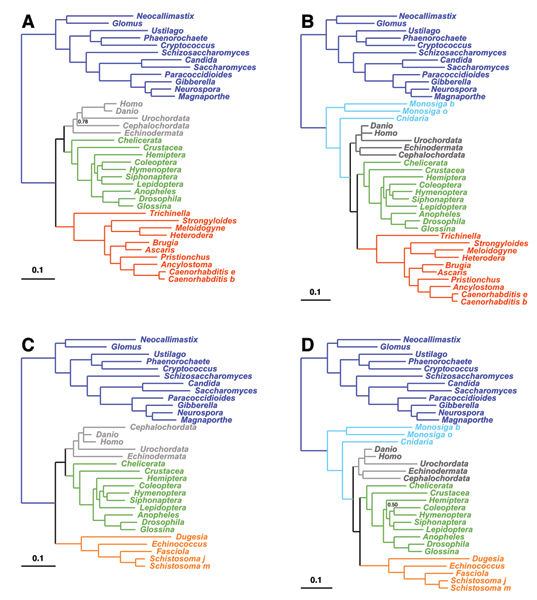
Posterior majority-rule consensus trees obtained under WAG+F+Γ, for the Meta1 data set, using four different taxon configurations: ingroup includes 5 deuterostomes (grey) and 10 arthropods (green), as well as 10 nematodes (red, A and B) or 5 platyhelminths (orange, C and D). Outgroup taxa comprise 12 fungi (dark blue), alone (A and C), or together with 2 choanoflagellates and a cnidarian (light blue, B and D). Posterior probabilities are displayed only when strictly lower than 1.

As observed previously [1,9,42], under WAG, the position of nematodes is strongly dependent on the outgroup (figure 1A,B): when a distant one (fungi) is used, nematodes are found at the base of the coelomates (arthropods and deuterostomes), whereas they are sister-group of arthropods (together called Ecdysozoa [15]), when two choanoflagellates and a cnidarian are added to the outgroup. These mutually contradictory positions for the nematodes are both supported with posterior probability 1, which makes the overall pattern a clear case of systematic artefact. As previously suggested [9], the Coelomata positioning, although supported by several recent large-scale phylogenomic analyses [5,6], might be an artefact due to an attraction of the fast-evolving group of nematodes by the long branch separating the ingroup and the outgroup, leading to an apparent basal emergence of nematodes. According to this interpretation, adding intermediate taxa to the outgroup has the effect of breaking this long branch, resulting in the presumably correct positioning of nematodes, within protostomes. A similar behavior is observed for platyhelminths (figure 1C,D): they are basal if the tree is rooted with fungi, whereas they are sister-group of arthropods, forming the clade of Protostomes, in the presence of choanoflagellates and the hydra.

**Figure 2:**
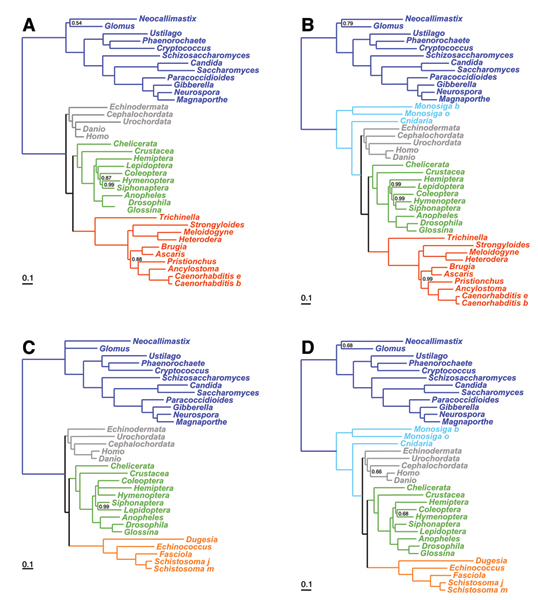
Posterior consensus trees obtained under CAT+F+Γ. Taxon sampling and color-codes are as in figure 1.

In contrast, under the CAT model (figure 2), both nematodes and platyhelminths keep their sister-group relationship with arthropods in all cases, even with the most distant outgroup. Therefore, in these two cases, CAT does not lead to mutually contradictory conclusions such as those proposed by WAG. Furthermore, it yields the topology that we expect if the interpretation in terms of LBA is correct. All these results are independently recovered for both halves of the alignment, except for the monophyly of deuterostomes, which was recovered with a low posterior probability (0.2) in one case (supplementary material). Taken together, they suggest that CAT is more robust to LBA than WAG.

### Model comparison by cross-validation

As a measure of model fitness, we evaluated the predictive power of CAT and WAG by cross-validation between the two datasets Meta1 and Meta2. For each model, the parameters (including the topology and the branch lengths) were learnt on one of the two datasets, and used to compute the likelihood of the other dataset. As is usual in a Bayesian Monte Carlo framework, the likelihood of the testing set is averaged over a sample obtained by MCMC from the posterior distribution under the learning set.

In the case of nematodes (table 1), CAT gave a higher cross-validated log-likelihood than WAG, indicating that it offers a better description of real data. Similar results were obtained when platyhelminths were used instead of nematodes (data not shown). Note that, to compute the cross-validated likelihood under CAT, we have made an approximation (see methods), but one which leads to an underevaluation of the cross-validation score of CAT. Since in all cases, CAT turns out to have the best score, this approximation does not invalidate our conclusions.

These results confirm our previous observations based on Bayes factor evaluations [31,41], showing that, in most instances, CAT offers a better statistical fit than WAG. Note that we previously found a few cases where CAT was not the best model [41], but this may be due to the small size of the single-gene datasets used in that former study, whereas in the present case, the very large number of columns makes it probably easier for a parameter-rich model such as CAT to achieve good performances.

### A posterior predictive saturation analysis

Part of the better fit of CAT may come from its higher ability to correctly detect sequence saturation, which would explain its greater robustness to LBA artefacts. This interpretation raises the issue of whether CAT offers a *sufficient *account of saturation. In this respect, comparative evaluations such as the cross-validatory comparison performed above, do not offer in themselves any measure of the absolute goodness-of-fit of the models under scrutiny, a problem usually referred to as model assessment [43].

The rationale for assessing a model is usually based on the following argument: when a model is adequate, that is, when it correctly describes the true evolutionary process, then the true data should be indistinguishable from data simulated under this model. We can thus check the adequacy of a model by actually performing simulations, and comparing the value of a pre-defined summary statistic evaluated on the true data (observed value), with the distribution obtained for this statistic under the simulated replicates (null distribution). A significant deviation between the observed value and the null distribution will indicate a model-misspecification problem.

The outcome of the goodness-of-fit test depends on the chosen statistic. Usually, one chooses a statistic that is meant to be particularly sensitive to those patterns in the data that we are interested in, or that are thought to play a fundamental role in the estimation procedure. In the present case, we chose two statistics that directly measure what we take to be the main cause of systematic artefacts, namely, sequence saturation. Specifically, we measure the number of substitutions (*n*), and the level of homoplasy (*h*), defined as the mean number of homoplasies (convergences and reversions) per site (see methods). We only considered the case of the nematodes, using the most distant outgroup (fungi), and performed the test under fixed topology, which we successively set to the Coelomata tree favored by WAG, and to the Ecdysozoa topology preferred by CAT.

**Figure 3:**
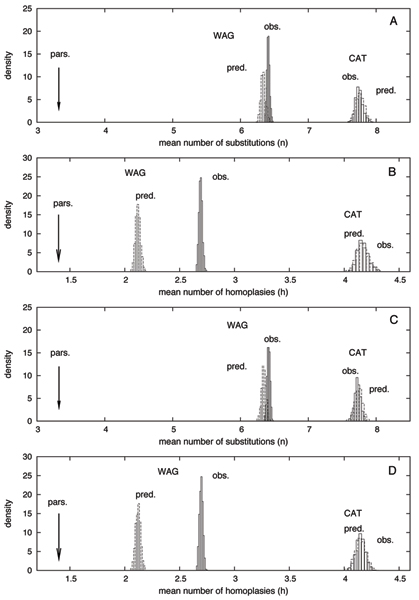
Posterior predictive statistical tests: Maximum Parsimony (pars. arrow), posterior distribution (obs. solid lines), and posterior predictive distribution (pred. dashed lines) of two statistics, *n*, the mean number of substitutions per site (A and C), and *h*, the mean number of homoplasies per site (B and D), under the Coelomata (A, B) and the Protostomia (C, D) hypotheses. The dataset is Meta1, with nematodes, and fungi as the only outgroup.

Under WAG, the observed (posterior) mean number of substitutions per site, *n*, is high, reaching 6.62 per site under the Coelomata tree (figure 3A). This is nearly twice as much as the MP estimate (3.53 per site), indicating that WAG recognizes a high level of sequence saturation in the absolute. Note however that the posterior mean value of *n *is even higher under CAT, up to 7.77 per site (figure 3A). In the two cases, the predictive distribution of *n *is close to the observed distribution (figure 3A), which is expected, since the length of the branches are free variables, which can adjust so as to match the observed and the predictive number of substitutions.

The posterior mean number of homoplasies per site *h *is also high under WAG (2.82 under the Coelomata hypothesis, figure 3B), and again, significantly higher than the MP estimate (1.397). However, the predictive mean number of homoplasies is much lower than the observed value (2.20 homoplasies per site). The difference, Δ*h *= 0.63, is large compared to the width of both the observed and the predictive distributions (figure 3B), indicating a clear lack of adequacy of WAG. In contrast, in the case of CAT, the posterior mean number of homoplasies, which is higher than under WAG, does not seem to significantly deviate from the observed distribution (mean of 4.18 per site for both observed and predictive, figure 3B). This indicates that, in contrast to WAG, CAT correctly accomodates the saturation patterns of sequences. Note that the deviation observed in the case of WAG is not simply accounted for by a difference in the observed and predicted number of substitutions (Δ*n *= 0.06, figure 3A), which suggest that the lack of adequacy of WAG lies in the way the substitutions are distributed either over the sites, over the branches, or over the 20 states. Since CAT and WAG differ only by the way they handle the amino-acid replacement processes, and otherwise, assume the same patterns of rate variations across sites, and ignore heterotachy, it seems likely that the observed deviation mainly lies in the way substitutions are distributed over the 20 states.

Similar results were obtained on the Ecdysozoa tree (figure 3C and 3D). Altogether, these observations confirm that the data are indeed saturated, to an extent that CAT not only better evaluates than WAG (the mean posterior saturation level is higher), but also, correctly anticipates (it matches the posterior predictive saturation level).

### Effective size of the amino-acid alphabet

What exactly makes CAT more able to detect homoplasy? As mentioned in the introduction, when looking at protein alignments, only a restricted subset of amino-acids is usually found at a given site. In accordance with this observation, under the CAT model, most sites are indeed inferred to evolve under highly peaked amino-acid profiles (figure 3 in [31]), that give a significant probability to only a few amino-acids. Importantly, these restrictions are encoded in the *stationary *probabilities (equilibrium frequencies) of the site-specific amino-acid replacement processes, and thus, they will have an influence even in the long run, after many substitutions. In contrast, in standard one-matrix models, such as WAG, the site-specific biochemical preferences are mediated mostly by the relative exchangeability parameters, and not by the stationary probabilities, which are in general close to, if not set equal to, the global empirical frequencies. Since the relative exchangeabilities only encode the *transient *behavior of the substitution process, in the long run, the same broad amino-acid profile is then inevitably expected at all positions under such matrices.

**Figure 4:**
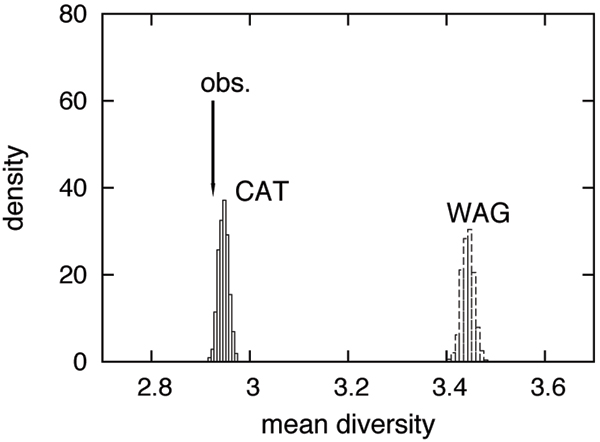
Posterior predictive analysis of the mean number of distinct residues observed at each column of the alignement (mean diversity). The analysis was done on Meta1, using the nematode/fungi taxon configuration.

To measure the impact of this difference between WAG and CAT on real cases, we performed a posterior predictive analyis of the mean number of different amino-acid per columns. As shown in figure 4, the observed mean number of distinct residues per column (or *biochemichal diversity*) is 2.93 on Meta1. Under WAG, the predictive biochemical diversity is significantly greater, of about 3.45 (*p *< 0.001), which means that WAG is strongly rejected for its inability to account for the site-specific biochemical specificities observed in real data, at least in the present case. In contrast, the biochemical diversity predicted by CAT (2.95) is much closer to the observed value (2.93), although CAT is also rejected (*p *= 0.007) by this posterior predictive test.

These spread-versus-peaked tendencies of WAG and CAT probably have a direct influence on the way these two models deal with sequence saturation. Essentially, if most sites undergo repeated substitutions among two or three amino-acids, then the true probability for a site to display the same state independently in two non-related species is only about one-third to one-half. If, however, one assumes that all 20 amino-acids can be observed at any position, as do standard empirical matrices, this probability is estimated to be much lower, as low as 1/20 if all amino-acids are considered as equally frequent. Under non-uniform equilibrium frequencies (e.g. empirical), this probability is higher, but still quite low. In contrast, models acknowledging site-specific restrictions of the amino-acid alphabet, as does CAT, can in principle correctly estimate this probability. This in turn implies that, in a real phylogenetic analysis, whenever two taxa will display the same state at the same site, CAT will be much more ready to interpret this observed identity as a homoplasy, rather than as a shared derived character. Thanks to this phenomenon, CAT is inherently more robust against homoplasies than WAG, which may be the reason why it does not produce the Coelomata artefact.

To get a more quantitative view of this phenomenon, we computed an index based on information-theoretic arguments, and meant as a measure of the effective number of amino-acids implied by a model of evolution (see methods). This index measures the effective size of the amino-acid alphabet. In the case of WAG, it is defined globally, over the alignment, and is here equal to 17.74. Under CAT, it is site-specific, and its average value over the alignment is equal to 4.35. By taking the inverse of these two numbers, one can estimate the probability of homoplasies, under complete saturation, at 0.23 for CAT, versus 0.06 for WAG.

**Figure 5:**
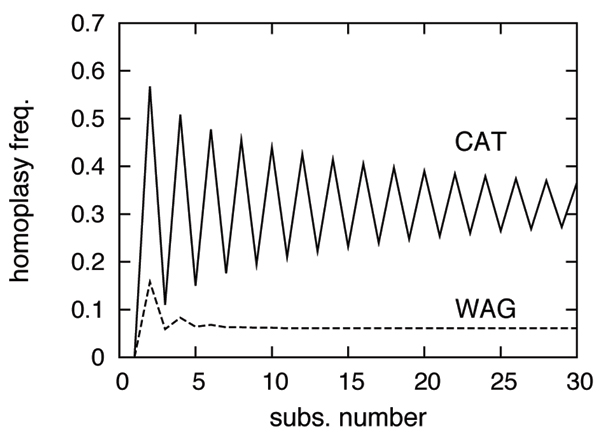
Average probability of return to the initial state, under WAG (dashed lines) and CAT (solid lines), as a function of the number of substitutions.

These estimates are only valid at stationarity. To know what happens at lower saturation, we measured the average frequency at which the substitution process returns to its initial state after 2, 3, *n *substitution events, under each model (figure 5). Since the process is reversible, reversions and convergences are equivalent, and we are thus computing the *n-step homoplasy probability*, i.e. the posterior predictive average probability that, at a given site, two species separated by *n *> 0 substitutions along the tree will be found in the same state.

As can be seen from this figure, the *n*-step homoplasy probability of WAG reaches the stationary state very rapidly, and then stays at a very low level (around 0.07). Thus, on the average, the effect of the exchangeability parameters of the matrix are damped after only 4 to 5 substitutions, after which the probability of the final state is essentially dictated by the stationary probabilities of the process. In contrast, under CAT, the *n*-step homoplasy probability is always higher than under WAG, and remains high (at around 0.32) even for large values of *n*. Note that, in both cases, the stationary value of the homoplasy level is close to that predicted above from the effective size of the alphabet, which confirms the fundamental link between the spread of the stationary profile(s) under each model, and how these models acknowledge sequence saturation.

### Impact of the effective size on the LBA artefacts

To more directly test the connection between the effective size of the amino-acid alphabet and the LBA problem, we performed a phylogenetic estimation under two overly simple models. At one extreme, we used the equivalent of the Jukes and Cantor model [44] for amino-acids, assuming all 20 amino-acid to be equally likely at each site. Under this model, the size of the amino-acid alphabet is maximal, and we therefore expect a strong under-evaluation of the true probability of homoplasies, and thus a higher sensitivity to LBA (an expansion of the Felsenstein zone) in this case. We call this model the "large-orbit" model (since it takes a long time, on average, for the process to revisit the same state). At the other extreme, we devise another flat model, but now, such that the set of allowed amino-acids at a given site is equal to the set of observed amino-acids at that site. In other word, we constrain CAT so as to give each site its own profile, which is fixed and gives a probability 1/*k *to each of the *k *amino-acids observed at the corresponding column, and a probability 0 to all other, non-observed, amino-acids. This "small-orbit" model should be the most skeptical among the flat models in interpreting shared character states as synapomorphies, and therefore, should not produce the artefact. Both models assume gamma-distributed rates across sites. Note that the "small-orbit" configuration is not a bona-fide statistical model, as it amounts to assuming that non-observed states at a given position would never be observed if new taxa were to be added to the alignment. Nevertheless, this toy-model is useful for measuring the impact of the change of the size of the alphabet, everything else remaining the same.

We analyzed the two data sets Meta1 and Meta2 using these two settings, and under the four taxon configurations. The results are concordant with our expectations: the large-orbit model yields the same mutually contradictory trees as WAG, whereas the small-orbit configuration leads to the same conclusions as CAT, i.e. both platyhelminths and nematodes were found sister group of arthropods, irrespective of the choice of the outgroup. This strongly suggests that the size of the amino-acid alphabet effectively accessible at each site is the dominant factor accounting for CAT's robustness against long branch attraction artefacts.

## Conclusion

We demonstrated that site-specific amino-acid replacement patterns are a crucial aspect of protein evolution, which is not correctly accounted for by the WAG empirical matrix. This particular model-misspecification problem is probably the major reason for the sensitivity of WAG to LBA artefacts.

In contrast, explicitly accounting for site-specific amino-acid stationary probabilities seems to be efficient at detecting saturation, and in some cases (figure 2), results in a complete disappearance of artefacts previously observed when using standard models.

Empirical matrices other than WAG were not tested in this work, nor was the behavior of the most general site-homogeneous reversible model (GTR) investigated. However, it should be stressed that all these alternatives, like WAG, encode amino-acid specificities in the relative exchangeability parameters. As we mentioned above, and as has been noticed previously [38], these relative exchangeabilities have an influence only on the short-term behavior of the amino-acid replacement process, whereas the amino-acid patterns observed in protein alignments are probably the result of site-specific selective forces, and are thus expected to be observed also in the long-term. Hence, we think that there is a logical flaw in the very idea of encoding biochemical realism into a single matrix. In practice, for all such matrices, there will always be a saturation level for which the probability of the states observed at a site is dominated by the global stationary probabilities, and thus, for which the model will underestimate the true saturation level.

Concerning the site-heterogeneous models, our main focus was on CAT, a mixture model that we proposed previously, but other more simple models accounting for site-specific amino-acid preferences should also display a similar robustness against saturation and LBA artefacts. For instance, even the small-orbit pseudo-model investigated above was able to overcome the two artefacts investigated in this paper. We also observed that constraining the total number of categories of CAT to be as low as 10 was sufficient to obtain the correct tree in all cases (not shown). Our feeling is that simple mixture models, based on an array of a fixed, and low, number of pre-specified empirical amino-acid profiles will be the best compromise between robustness and computational efficiency.

The substitution processes are also likely to be site-heterogeneous at the nucleotide level, for coding as well as for non-coding sequences. Mixture models have also been implemented at this level [32], and they may display a similar robustness agains systematic artefacts. Note, however, that, given the relatively small size of the nucleotide alphabet, the actual level of saturation may not be as strongly under-evaluated by standard one-matrix models in the case of nucleotides sequences, as it is for proteins.

Finally, it should be stressed that there might be other kinds of model violations also causing LBA artefacts, such as across time rate variation (heterotachy) [22], or global compositional biases. A model handling all these features should ultimately be considered, and may offer a more satisfactory answer to the problem of tree reconstruction artefacts.

## Methods

### Data: gene and taxon subsampling

We used the same data as in previous analyses [1,9,42]. These are made of the sequences of 146 genes, from 49 taxa. We divided this dataset into two subsets by random and independent assignment of each gene. The two resulting alignments, hereafter called Meta1 and Meta2, are of approximately the same size (17,807 and 17,564 positions, respectively). Splitting the dataset into two halves was motivated by the cross-validation approach explained below, in which models are fitted on one half, and tested for their predictive power on the other. It also makes the analyses more manageable, in terms of CPU time. In addition, the congruence of the topologies estimated on each half offers a rough qualitative estimate of the robustness, without performing a prohibitive bootstrap analysis.

We considered the following taxa combinations: the ingroup (Bilateria) includes 5 deuterostomes, 10 arthropods, and either 10 nematodes or 5 platyhelminths. The outgroup is composed of (1) 12 fungi, or (2) 12 fungi, 2 choanoflagellates and 1 cnidarian. This defines a series of 4 taxa subsets which, when applied to the 2 datasets Meta1 and Meta2, yield a total of 8 datasets.

### Models and implementations

We considered two alternative models of amino acid replacement: the WAG matrix [39], with stationary probabilities set to the empirical frequencies, and a site-heterogeneous model, CAT [31]. Briefly, CAT is a mixture model based on a Dirichlet process. It has a free number *L *of categories controlled by a hyperparameter *ε*. To each category corresponds a profile *π*_*l*_, which is a probability vector over the 20 amino-acids. The evolutionary process defined by this category is then similar to that proposed by Felsenstein [45], i.e. a Poisson process distributing events across time, so that at each event, a new state (possibly equal to the current one) is drawn at random from the probability profile.

In our initial implementation, the profiles were drawn from a uniform distribution [31]. To increase the model's flexibility, we now use a general Dirichlet distribution controlled by two hyperparameters, *π*_0 _and *δ*. The probability vector *π*_0 _defines the mean of the distribution, whereas *δ *controls the dispersion around that mean, with small values corresponding to a large dispersion. In addition, the distribution of site-specific relative rates of substitution are not anymore a Dirichlet, but as in more classical phylogenetic models, a gamma distribution of mean 1 and shape paremeter *α *[25]. Both *α *and *δ *are endowed with an exponential prior of mean 1. The priors over the Dirichlet process hyperparameter *ε *and over *π*_0 _are uniform.

Our implementation is similar to the previous one, except that it now includes conjugate Gibbs sampling operators, which yield a much more efficient mixing than the algorithms previously described. This implementation also includes the WAG model. The software is available from our web site [47].

Under CAT, Markov chains were run for 12,000 cycles, discarding the first 4,000 points, and then saving a point every 10 cycles. Under WAG, chains were extended for 6,000 cycles, discarding the first 2,000 points. Each cycle combines general Metropolis-Hastings updates such as topological moves (a total of 100 per cycle, including node-sliding, Local, Global and TBR moves), or updates of the alpha parameter, together with model-specific updates of the parameter vector (in the case of CAT). For each experiment, we performed two independent runs under CAT, starting from a random topology. In the case of WAG, we performed one run with our program, and another one using MrBayes [48]. One run takes about 15 days on a hyperthreaded Xeon (3.06 GHz).

In all cases, the two independent experiments always lead twice to the same tree except for the exact relationships among deuterostomes, which showed some instability, as well as the position of hymenopterans. The average standard deviation of bipartition frequencies between the two runs ranged from 0.01 to 0.05, depending on the datasets.

### Cross-validations

In previous studies [31,41], we compared models by Bayes factor evaluation [49]. However, Bayes factors involve heavy numerical estimation procedures, which cannot be applied to the present large data sets. Instead, we will use cross-validation [50].

A good model should be able to predict future data. Accordingly, the general idea of cross-validation is to learn the parameters of the model on one half of the available data (*D*_1_), and then test the predictive power of the model on the other half (*D*_2_). Formally, this predictive power is measured by the marginal probability of *D*_2_, given our knowledge of *D*_1_:

*p*(*D*_2 _| *M*, *D*_1_) = ∫*p*(*D*_2 _| *θ*, *M*)*p*(*θ *| *D*_1_, *M*)*dθ*,     (1)

with *θ *standing for the set of all parameters of the model. Note that, since the models are tested on data that they have not observed during the learning step, over-fitting or self-reinforcement artefacts are alleviated, and the values obtained for different models can directly be compared, without having to explicitly account for each model's dimensionality.

Equation 1 is an average of the likelihood of *D*_2 _over the posterior distribution under *D*_1_. As such, it can be approximated by the usual Monte Carlo procedure. Specifically, having obtained a collection of values ()_*k*=1..*K *_drawn from *p*(*θ *| *M*, *D*_1_), one has:



The same procedure is applied in the other direction (i.e. sampling from the posterior under *D*_2_, and evaluating the mean posterior likelihood of *D*_1_).

As in usual mixture models, the site-specific likelihoods under CAT are weighted sums over the available categories. In our implementation, however, these sums are not performed explicitly, but are implicit in the MCMC sampling: sites are each allocated to one among the available categories, a configuration which is regularly updated by Gibbs sampling. Conversely, the weights of the categories have been integrated away, conditional on the categories' occupation numbers (i.e. the number of sites allocated to each) [31]. Nevertheless, to compute the cross-validation likelihoods, these sums are now needed. Specifically, suppose that a parameter configuration  has been sampled from the learning posterior distribution (given *D*_1_). Under this parameter value, *L *categories are defined, characterized by their *L *profiles *π *= (*π*_*l*_)_*l*=1..*L*_, and their occupation numbers *η *= (*η*_*l*_)_*l*=1..*L*_, with ∑_*l*=1..*L *_*η*_*l *_= *N*_1_, the number of sites of *D*_1_. Then, given these specifications, and given the current value of the Dirichlet process hyperparameters *ε*, *π*_0 _and *δ*, the probability of a given column pattern is given by the conditional formula [31,51]:



where the prior on *π*, *p*(*π *| *π*_0_, *δ*), is the generalized Dirichlet distribution mentioned above. The sum over *l *in the right-hand side of equation 3 is the likelihood at site *i *averaged over all possible affiliations of site *i *to each of the available profiles. The last term, the integral over *π*, corresponds to the probability of the current column pattern (*C*_*i*_) to be described by its own profile, and not by any of those proposed by the current point *θ*_1_. In our present computations, we neglect this last term, and compute only the average likelihood over already existing profiles. We thus obtain a slight under-estimation of the cross-validation likelihood in this case.

### Posterior predictive analyses

Given a statistic *s*, posterior predictive hypothesis testing consists in comparing the observed value of *s *on the available data *D*, *s*_*obs *_= *s*(*D*), with the distribution of *s *under replicates *D*_*rep *_simulated from the posterior predictive distribution:

*D*_*rep *_~ *p*(*D*_*rep *_| *D*, *M*).     (4)

A significant deviation of the posterior predictive distribution of the statistic from the observed value means that the model is not able to recreate, upon simulation, the patterns captured by *s *on observed data [52,53], and is thus rejected.

Replicates are obtained by first drawing a series of values (*θ*^(*k*)^) from the posterior distribution *p*(*θ *| *D*, *M*). Then, for each *k*, replicates  are simulated based on the parameter value *θ*^(*k*)^. Once this is done, the statistic is evaluated on each replicate, *s*^(*k*) ^= *s*(), and the resulting distribution is compared with the observed value *s*_*obs*_. An estimate of the posterior predictive p-value is obtained by computing the frequency at which *s*^(*k*) ^> *s*_*obs*_, and rejection is assessed by checking whether this frequency is below a given threshold [52]. Here, we used the standard threshold of 0.05.

We investigated several statistics. First, to evaluate how well the models reproduce the specificities of the column patterns, we measured the mean number of distinct residues observed at each column (which we call the *biochemical diversity*). Second, we measured two statistics depending on the ancestral reconstruction of the characters along the tree, namely, the number *n *of substitutions over the whole tree, and the number *h *of homoplasies. For a given site *i*, and given an ancestral state reconstruction, we count the number of substitutions observed in this reconstruction towards each amino-acid *j*, and add one to this number if the state at the root is also *j*. This number, which we denote by *m*_*ij*_, can be understood, from a cladistic point of view, as the number of *evolutionarily independent *times site *i *evolved to state *j*, according to the chosen ancestral reconstruction. The number of homoplasies observed at site *i*, under the current mapping, is then defined to be equal to:



Note that convergences and reversions may transform into each other, upon rerooting of the tree. Both are accounted for by this number, which is invariant with respect to the position of the root. We then define the mean number of homoplasies per site as .

The statistics *n *and *h *depend on the tree topology, which was considered fixed. In addition, they also depend on the ancestral state reconstruction, which is not actually observed, and has to be inferred from the data. We thus proceeded by stochastic character mappings, as proposed by Nielsen [54]. According to this stochastic version of the posterior predictive test, the observed value of the statistic is now the posterior distribution under the observed data. For simplicity, we will still call it the *observed *distribution. It is obtained as follows: for a given parameter value (*θ*^(*k*)^), a stochastic mapping is sampled from the posterior distribution given the states observed at the leaves, and using the procedure described by Nielsen [54]. The statistic (e.g. the total number of substitutions *n*) is computed on this mapping, which yields one realization of *n*. This procedure is repeated for each (*θ*^(*k*)^), *k *= 1..*K*, so as to yield a distribution of values of *n*, meant as an estimate of the observed distribution. The null (*predictive*) distribution is obtained in exactly the same way, except that the mappings are now simulated without constraining the states at the leaves. This stochastic procedure is Bayesian in spirit, as it accounts for the uncertainty about the past substitution events on which our two statistics, *n *and *h*, are based. On the other hand, there is no well-defined p-value anymore (to our knowledge, none has been described in the literature). Nevertheless, the two distributions can still be visually compared, which offers a useful, albeit qualitative, way of checking the model's behavior.

### Effective size of the amino-acid alphabet and probability of homoplasies

We wish to evaluate how much the equilibrium frequency profile of a substitution model is spread over the 20 amino-acids. A common measure of how much a probability distribution is spread over its definition domain (here the 20 amino-acids) is the Shannon entropy:



Here, we define the *effective number *of amino-acids, (or the effective size of the amino-acid alphabet) implied by the stationary distribution *π *as the exponential of its Shannon entropy. In particular, for a distribution that gives a probability 1/*k *to each of a subset of *k *amino-acids, and 0 to all other amino-acids, the effective size is equal to *k*. As a convenient summary, for a given model, the effective size was averaged over the MCMC (and over all sites in the case of CAT).

We also computed the frequency at which the substitution process returns to its initial state after *n *substitution events. Note that since the process is reversible, reversions and convergences are equivalent, and thus, as a function of *n*, what we are computing is the *n-step homoplasy probability*, i.e. the probability that at a given site, two species separated by *n *> 0 substitutions along the tree will be found in the same character state. For a given configuration of the model, these frequencies can be computed analytically.  They were then averaged over sites, states, and the posterior distribution.

## Authors' contributions

NL made the implementation of the method, performed the run of all the experiments, and wrote the initial version of the manuscript. HB provided the alignments, participated in the run of the experiments, and contributed to the writing. HP contributed to the drafting of the manuscript. All authors read and approved the final manuscript.

## Figures and Tables

**Table 1:** Cross validation posterior mean log likelihoods under WAG and CAT. The computation was done using both outgroup configurations (lines), and in both directions, i.e. using either dataset as the learning and as the testing set (columns).

	*M*_1 _→ *M*_2_		*M*_2 _→ *M*_1_	
	CAT	WAG	CAT	WAG

nematodes fungi	-434,549	-448,857	-469,694	-485,718
nematodes fungi/choano/hydra	-442,828	-480,516	-478,880	-521,542
